# Mildly elevated succinylacetone and normal liver function in compound heterozygotes with pathogenic and pseudodeficient *FAH* alleles

**DOI:** 10.1016/j.ymgmr.2017.12.002

**Published:** 2017-12-27

**Authors:** Hao Yang, Francis Rossignol, Denis Cyr, Rachel Laframboise, Shu Pei Wang, Jean-François Soucy, Marie-Thérèse Berthier, Yves Giguère, Paula J. Waters, Grant A. Mitchell, the Québec NTBC Study Group

**Affiliations:** a Division of Medical Genetics, Department of Pediatrics, CHU Sainte-Justine, Université de Montréal, Montréal, Québec, Canada; b Service de Génétique médicale, Département de Pédiatrie, Centre hospitalier universitaire de Sherbrooke (CHUS), Sherbrooke, Québec, Canada; c Service de Génétique médicale, Département de Pédiatrie, CHU de Québec-Centre hospitalier de l'Université Laval (CHUL), Québec, Canada; d Programme québécois de Dépistage Néonatal Sanguin, CHU de Québec-Université Laval, Québec, Canada

**Keywords:** Tyrosinemia, Hypersuccinylacetonemia, Fah, Fumarylacetoacetate hydrolase, Nitisinone, Pseudodeficiency

## Abstract

**Background:**

A high level of succinylacetone (SA) in blood is a sensitive, specific marker for the screening and diagnosis of hepatorenal tyrosinemia (HT1, MIM 276700). HT1 is caused by mutations in the *FAH* gene, resulting in deficiency of fumarylacetoacetate hydrolase. HT1 newborns are usually clinically asymptomatic, but have coagulation abnormalities revealing liver dysfunction. Treatment with nitisinone (NTBC) plus dietary restriction of tyrosine and phenylalanine prevents the complications of HT1

**Observations:**

Two newborns screened positive for SA but had normal coagulation testing. Plasma and urine SA levels were 3–5 fold above the reference range but were markedly lower than in typical HT1. Neither individual received nitisinone or dietary therapy. They remain clinically normal, currently aged 9 and 15 years. Each was a compound heterozygote, having a splicing variant in *trans* with a prevalent “pseudodeficient” *FAH* allele, c.1021C > T (p.Arg341Trp), which confers partial FAH activity. All newborns identified with mild hypersuccinylacetonemia in Québec have had genetic deficiencies of tyrosine degradation: either deficiency of the enzyme preceding FAH, maleylacetoacetate isomerase, or partial deficiency of FAH itself.

**Conclusion:**

Compound heterozygotes for c.1021C > T (p.Arg341Trp) and a severely deficient FAH allele have mild hypersuccinylacetonemia and to date they have remained asymptomatic without treatment. It is important to determine the long term outcome of such individuals.

## 1 Introduction

**Fig. 1:**
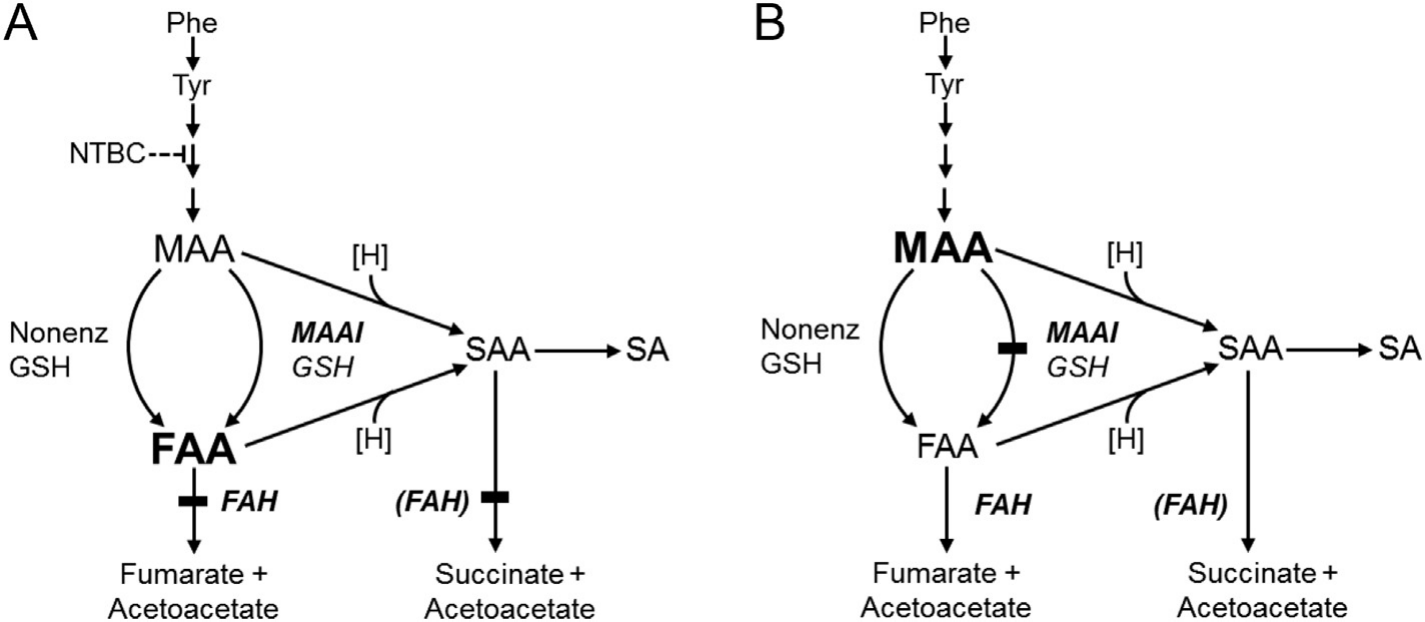
The degradation pathway of phenylalanine and tyrosine, showing the different metabolite patterns predicted in FAH and MAAI deficiencies. Maleylacetoacetate (MAA), fumarylacetoacetate (FAA), succinylacetoacetate (SAA) and succinylacetone (SA) are reported to be toxic [1]. The first three are reactive and labile, and have not been accurately measured in tyrosinemic liver. The relative toxicities of FAA and MAA are not known, but the clinical severity of classical HT1 [1], [3] contrasts with the apparently mild natural history of MAAI deficiency [9]. By extension, FAA may be more toxic. (A) In mild hypersuccinylacetonemia (MHSA) due to FAH deficiency, as in untreated p.R341W/HT1 compounds or in classical HT1 that is treated with suboptimal doses of NTBC, the concentration of FAA is predicted to be high, and that of MAA, normal or mildly elevated. (B) At a similar level of plasma SA, MAAI deficiency is predicted to cause higher MAA in liver than FAH deficiency, but low levels of FAA.

A marked increase in succinylacetone (SA) is regarded as pathognomonic for hepatorenal tyrosinemia (tyrosinemia type I, HT1, MIM 276700) [1], [2], a severe autosomal recessive disease that causes hepatic failure, cirrhosis, liver cancer, renal tubulopathy, rickets and severe porphyria-like neurological crises. Medical treatment of HT1 is highly effective. All of these complications are preventable by diagnosis and treatment with nitisinone (NTBC) plus dietary therapy, if this treatment is started in the first month of life [3]. Sensitive newborn screening is therefore essential for optimal treatment of HT1. HT1 results from deficiency of fumarylacetoacetate hydrolase (FAH), encoded by the *FAH* gene. FAH is the last enzyme of the degradation pathway of phenylalanine (Phe) and tyrosine (Tyr, Fig. 1).

SA is a sensitive and specific marker for the screening and diagnosis of HT1 [4]. HT1 is included in the Recommended Uniform Screening Panel [5] and SA is recognised as the best primary screening marker for HT1. Tandem mass spectrometry-based detection of SA [6] is increasingly used for population newborn screening. For confirmatory diagnostic testing, stable isotope dilution assays for SA permit accurate SA measurement, with sensitivity in the low nanomolar range [7].

The longest-running neonatal screening program for HT1 is in Québec, Canada, where HT1 is prevalent because of a genetic founder effect [8]. The program began in 1970. Bloodspot SA has been the first tier screening marker since 1996. To date, two conditions that cause mild hypersuccinylacetonemia have been identified in individuals referred as “screen-positive” by the program. Some of these individuals have deficiency of maleylacetoacetate isomerase (MAAI), the enzyme preceding FAH in tyrosine degradation [9].

The other two individuals with mild hypersuccinylacetonemia, described in this article, have partial FAH deficiency due to compound heterozygosity for a pseudodeficient *FAH* allele and a *FAH* allele with severely deficient function. *FAH* pseudodeficiency was reported three decades ago [10], as an incidental finding in small numbers of clinically-normal individuals with unexpectedly low FAH activity [11]. Individuals who were compound heterozygotes of the single nucleotide variant c.1021C > T (p.Arg341Trp) in *trans* with an allele that confers severe FAH deficiency [11] (henceforth designated the *p.R341W/HT1* genotype) were described as having normal SA levels in plasma and urine [10], but loading studies with oral tyrosine and intravenous homogentisate resulted in a brief elevation of SA to about 2 μmol/L in one p.R341W/HT1 individual [10]. In cultured fibroblasts, cells from an individual reported to be homozygous for p.R341W yielded low immunoreactive FAH protein levels and about 10% of normal activity [11]. Here we report that p.R341W/HT1 individuals have mild persistent hypersuccinylacetonemia and discuss the importance of this observation.

## 2 Methods and results

### 2.1 Clinical evaluation and treatment decisions in patients referred for hypersuccinylacetonemia

The current protocol for the clinical evaluation and treatment of newborns identified by screening for HT1 has been described [9], [12]. It includes clinical evaluation and followup in one of five academic medical centers in Quebec and diagnostic testing for SA at CHUS.

### 2.2 Diagnostic analysis of SA

The diagnostic assays for SA in plasma and urine, developed and performed at CHUS, involve the addition of ^13^C5-succinylacetone as internal standard, followed by oximation, extraction, derivatization and gas chromatography-mass spectrometric determination, as described [7].

### 2.3 Case descriptions

Both patients were referred for possible HT1 by the Quebec newborn screening program. Individual 1 (I-1) is a 15-year-old boy born at 37 5/7 weeks by spontaneous vaginal delivery after a medically-unremarkable pregnancy. His birth weight was 3335 g (70th centile). His Apgar scores were 9 and 10 at one and five minutes respectively. He is the second child of healthy non-consanguineous parents of French-Canadian descent. The family history is unremarkable. Physical examination and coagulation testing using prothrombin time and/or international normalized ratio (INR) were normal at the first evaluation.

Individual 2 (I-2) is a 9 year-old boy, the second child of a non-consanguineous couple of French-Canadian descent. After a medically-unremarkable pregnancy, delivery was induced at 37 weeks for deceleration of fetal growth. His birth weight was 2696 g (30th centile). His Apgar scores were 7, 9 and 9 at 1, 5 and 10 min, respectively. Family history was unremarkable. At the first evaluation, both physical examination and INR were normal.

In each case, the family opted for medical surveillance without nitisinone and dietary treatment. Growth, development, hepatic magnetic resonance imaging, abdominal ultrasound, plasma alpha-fetoprotein, INR, and plasma aminotransferases and bilirubin, verified serially in each case, have been normal. Fasting blood glucose levels have repeatedly been normal. Physical examination, in particular of the abdomen and the heart, has remained normal. Individual 1 was assessed twice by echocardiography, at one month of age and around the time of school entry, and this was normal each time. Individual 2 has not had formal cardiac ultrasound testing. Tyrosinemic neurologic crises [13] have not occurred.

### 2.4 Biochemical findings

SA levels in plasma have been mildly but consistently elevated (Table 1). In urine, SA was elevated in most samples (Table 1) but in 3/12 (25%) of the samples of I-1 and in 1/17 (5.9%) of those of I-2, the levels of SA fell within the reference range.

**Table 1:** Succinylacetone levels in plasma and urine of two p.R341W/HT1 individuals.

	Initial evaluation [median (range, n)]		Follow-up samples [median (range, n)]	
	Plasma SA (nmol/L)	Urine SA (μmol/mol Cre)	Plasma SA (nmol/L)	Urine SA (μmol/mol Cre)
Individual 1	107	93	58 (38–67, n = 5)	51 (23–102, n = 12)
Individual 2	138	59	111 (66–311, n = 21)	143 (17–368, n = 19)
HT1 patients^a^	39,689 (10,206–128,837, n = 23)	284,557 (59,921–1,195,273, n = 23)	NA	NA
Reference range	< 24	< 34	< 24	< 34

Abbreviations: Cre, creatinine; NA, not applicable.

a The HT1 patients represent the 23 most recently screened HT1 patients in Québec for whom adequate pretreatment plasma and urine specimens were available, tested as described by the reference laboratory (CHUS, Sherbrooke, Québec).

Blood and plasma levels of tyrosine have always been within the reference range for I-1. For I-2, blood spot tyrosine was 229 μmol/L at two days of age and 249 μmol/L at 11 days of age (reference values < 200 μmol/L); at one month of age, it was 171 μmol/L (reference range, 43–108). The significance, if any, of these mildly elevated values in the newborn period is unknown. After one year of age, all plasma and blood spot tyrosine levels of I-2 have been < 80 μmol/L (n = 15), within their respective age-related reference ranges.

### 2.5 Molecular analysis

Sanger sequencing of all exons and flanking intronic sequences of *FAH* in certified commercial laboratories showed that both individuals are compound heterozygotes. Both are heterozygous for the previously reported “pseudodeficient” variant c.1021C > T (p.Arg341Trp; 15:80472526 C/T, rs11555096) [11]. The other *FAH* allele of each individual contains a splice site mutation. I-1 has the common French-Canadian founder mutation, c.1062 + 5G > A (formerly designated IVS12 + 5 G > A [8]). I-2 has a previously undescribed variant, c.553 + 1G > A, situated at the + 1 position of the donor splice site of *FAH* intron 6. Applying the American College of Medical Genetics guideline criteria [14] this variant is classified as likely pathogenic because it alters invariant residues of a canonical splice site (PVS1) and it is rare, being absent from the ExAc data base (PM2).

## 3 Discussion

The two p.R341W/HT1 compound heterozygotes described in this article differ from classical HT1 in three important ways. First, clinically, they had normal liver function on initial evaluation and thereafter. In our experience, elevated international normalized ratio (INR) or prolonged prothrombin time (PT) are consistent findings in newborns with untreated classic HT1 [1]. This observation is useful for deciding upon the initial clinical approach, because INR and PT results are available on the day of the first clinical evaluation.

The second difference from classical HT1 patients relates to the profile observed on newborn screening. Both I-1 and I-2 were identified during a period when the newborn screening algorithm for second tier testing of mild hypersuccinylacetonemia called for two parallel steps: confirmatory SA determination in a repeat blood spot and detection of immunoreactive FAH protein in a blood spot. The samples of I-1 and I-2 were considered atypical because they had markedly reduced immunoreactive FAH protein but only marginally increased SA. The small elevations of SA of I-1 and I-2 in newborn bloodspots would likely not have been identified by most screening programs [4]. Of note, testing for immunoreactive FAH was discontinued in newborn screening in Québec, and is not recommended for general use in other populations because some variants with severely reduced activity can produce normal protein levels [15].

The third difference from HT1 is that both newborns also had only mild elevations of SA during follow-up, compared to classic HT1 patients. Here we refer to “mild hypersuccinylacetonemia”, to designate the 50-fold interval between the upper reference value for plasma SA used in Québec [7] (24 nmol/L, Table 1) and the thresholds used by many diagnostic laboratories [4], typically about 1 μmol/L. Such individuals therefore, even if identified by newborn screening in other jurisdictions, would probably be dismissed as false-positives following “normal” confirmatory testing results. We have observed mild hypersuccinylacetonemia in three conditions: individuals with MAAI deficiency, HT1 patients with subtherapeutic levels of NTBC and p.R341W/HT1 individuals (Fig. 1).

To date, all HT1 patients in the Québec NTBC Study have had pretreatment plasma SA values well above 1 μmol/L. However, in the era before treatment with NTBC and before routine molecular diagnosis of HT1, there were reports of relatively mild elevations of SA in the blood and urine of a small fraction of patients treated by diet alone [1], [9] and in at least one case, in amniotic fluid of an affected pregnancy [16]. Although the assays used in these reports lacked the sensitivity of current methods and although reports were not always based on repeated assays, it is possible that some HT1 patients may have only subtly elevated SA levels. For this reason, the Québec screening program has established a relatively low threshold for clinical referral of infants with hypersuccinylacetonemia. Accurate follow-up assessment of these individuals has been assured by the availability of the highly-sensitive SA assay method [7] for confirmatory testing.

p.R341W/HT1 individuals were initially described as having normal SA levels in plasma and urine [10] but I-1 and I-2 have consistently had mildly elevated levels. This apparent discordance is likely explained by the limit of sensitivity of the SA assay in the first description of p.R341W/HT1 compounds, 150 nmol/L [10]. This is insufficient to detect the mild elevations in these individuals (Table 1).

In newborns with mild hypersuccinylacetonemia and normal liver function, nontreatment with surveillance of biochemical and liver function is an option. This contrasts with classic HT1, for which rapid initiation of NTBC and dietary therapy is clearly the treatment of choice [3]. This treatment requires sustained daily effort and close surveillance to prevent nutritional deficiencies, hypertyrosinemia and corneal ulcers [17]. Also, learning problems and delayed development are reported in treated HT1 patients [18], [19], and NTBC and dietary treatments are expensive [20]. All families in Québec with mild hypersuccinylacetonemia due to a p.R341W/HT1 genotype or to MAAI deficiency have chosen non-treatment with ongoing monitoring of SA levels and of liver function and imaging. To date, all have retained normal liver function.

To date, the SA concentrations of p.R341W/HT1 individuals have been stable, and we have not detected any times, for example during episodes of catabolic stress, at which high SA levels or detectable liver dysfunction occurred. Such an occurrence is theoretically possible and if a p.R341W/HT1 individual did become symptomatic, we would consider this to be an indication for NTBC treatment, at least for the duration of the stress.

Three observations taken together suggest that p.R341W/HT1 compounds are asymptomatic. A small number of p.R341W/HT1 adults detected by coincidence were reported as asymptomatic, although detailed clinical descriptions were not provided [10]. Second, detailed evaluation of liver function in the two young p.R341W/HT1 individuals described in the present report has so far detected no abnormality. Third, population data suggest that most p.R341W/HT1 individuals are not identified. The c.1021C > T (p.Arg341Trp) allele accounts for 1.6% of *FAH* alleles in the ExAC database (http://exac.broadinstitute.org/), and for 2% in 516 healthy Norwegian volunteers [11]. At this allele frequency, p.R341W/HT1 individuals are predicted to be more prevalent at birth in most populations than classical HT1 patients. The fact that so few p.R341W/HT1 individuals have been reported suggests that such individuals are well and do not come to medical attention.

Empirical data on liver function of elderly p.R341W/HT1 compound heterozygotes could definitively resolve whether p.R341W/HT1 individuals have normal long term liver function,no increased risk of hepatocarcinoma and otherwise remain healthy. Genomic information is increasingly available and in the future, most p.R341W/HT1 individuals will likely be identified in this fashion rather than by metabolic screening. In contrast with the original publications, we document chronic hypersuccinylacetonemia in p.R341W/HT1 individuals. This situation presumably reflects elevated intrahepatic concentrations of FAA and related metabolites (Fig. 1). This cannot be assumed to be benign. It is important to establish whether liver pathology occurs in a series of such patients.

Conversely, if the p.R341W/HT1 genotype does allow normal lifelong liver function with low cancer risk, this observation would help to refine the treatment of HT1. Current guidelines for HT1 treatment recommend that SA levels be maintained within the reference range [21]. Available information does not define a threshold of plasma SA below which the risk of liver damage and hepatocarcinoma is not elevated. The pattern of toxic metabolite levels in the liver of p.R341W/HT1 individuals is likely to be similar to that of HT1 patients with subtherapeutic levels of NTBC. This contrasts with the other form of mild hypersuccinylacetonemia, due to MAAI deficiency (Fig. 1). This might lead to speculation that, if p.R341W/HT1 individuals could be convincingly shown to have normal liver health into late adult life, their SA levels might provide a biologically relevant upper limit that would be useful for assessing NTBC treatment of HT1 patients.

However, tyrosine metabolism is not predicted to be strictly identical in p.R341W/HT1 individuals and in HT1 patients who receive subtherapeutic doses of NTBC that only partially inhibit 4-hydroxyphenylpyruvate dioxygenase. In p.R341W/HT1 individuals, the catalytic capacity of FAH is moderately reduced, and in the presence of a normal flux of tyrosine degradation, this leads to mild accumulation of SA. In contrast, HT1 patients with subtherapeutic levels of NTBC have only partial inhibition of 4-hydroxyphenylpyruvate dioxygenase. It is felt that in these individuals, a small fraction of tyrosine trickles through to the later steps of tyrosine catabolism, from which its exit is arrested by the severe deficiency of FAH. At a given level of plasma SA, the kinetics and bioavailability of SA and other tyrosine metabolites may differ between the tissues of p.R341W/HT1 individuals and those of HT1 patients receiving insufficient NTBC treatment.

Even allowing for these possible differences, p.R341W/HT1 individuals provide the closest situation yet described in humans to that of partially treated HT1 patients receiving doses of NTBC that only partially inhibit 4-hydroxyphenylpyruvate dioxygenase. Furthermore, such individuals will be identifiable as genomic data becomes available for a large number of people. Knowledge of their long-term outcome will be useful for determining the optimal clinical approach to children with the p.R341W/HT1 genotype and to HT1 patients treated with nitisinone.

## Conflict of interest

The authors declare no conflict of interest.
